# A feasibility randomized controlled trial of culturally adapted Getting Better Bite-by-Bite (Ca-GBBB) intervention for individuals with eating disorders in Pakistan

**DOI:** 10.1186/s40337-024-01038-4

**Published:** 2024-07-17

**Authors:** Ayesha Khaliq, Amina Muazzam, Rafia Rafique, Tayyeba Kiran, Ameera Ahmed, Irfan Suleheria, Nasim Chaudhry, Nusrat Husain

**Affiliations:** 1 Pakistan Institute of Living and Learning, Karachi, Pakistan; 2 Lahore College for Women University (LCWU), Lahore, Pakistan; 3 Institute of Applied Psychology, University of the Punjab, Lahore, Pakistan; 4 Shaikh Zayed Hospital, Lahore, Pakistan; 5 Islam Medical and Dental College, Sialkot, Pakistan; 6 Grand Asian University, Sialkot, Pakistan; 7 Farooq Hospital, Lahore, Pakistan; 8 The University of Manchester, Manchester, UK; 9 Mersey Care NHS Foundation Trust, Liverpool, UK

**Keywords:** Eating disorders, Bulimia nervosa, Binge eating, Mental health, Bite by bite, Pakistan, LMICs, Cultural adaptation, Psychological intervention

## Abstract

**Background:**

Eating disorders (EDs) are serious mental health conditions that affect a person physically and psychologically. In the past, EDs were only recognized as a cultural phenomenon/societal by-product of the West. However, research evidence marks its presence in non-western countries also, including South Asia. This study aims to evaluate the feasibility and acceptability of a manualized psychological intervention called Getting Better Bite by Bite (GBBB) in individuals who screened positive on measures of EDs in Pakistan.

**Methods:**

The proposed study is a feasibility randomized controlled trial (fRCT). The study will be conducted at five sites across Pakistan: Karachi, Hyderabad, Lahore, Rawalpindi, and Multan to recruit a total of 80 participants. Eligible participants will be randomized to either (1) the intervention group; in which they will receive one-to-one sessions of GBBB along with routine care or (2) the routine care group; in which they will only have access to the routine care. We have received ethics approval by the National Bioethics Committee. The study is registered at clinicaltrials.gov (NCT05724394). The study team has received permission from recruitment centers: hospitals (i.e. the psychiatry department of public and private hospitals based in these cities), fitness centers (i.e., gyms), educational institutes (i.e., colleges and universities), and community settings (i.e. community health clinics). Self-referrals from General Practitioners and community settings will be accepted. The intervention manual has been translated into Urdu and a multidisciplinary team including service users has culturally adapted the content of intervention for local context.

**Discussion:**

This study will provide evidence on feasibility and acceptability of a culturally adapted intervention for individuals who screen positive on measures of EDs. The findings of this study will inform a fully powered Randomized Controlled Trial of the proposed intervention.

*Trial Registration*. The study is registered on clinicaltrials.gov (NCT05724394). Protocol version (1.0. 1st June 2022).

## Background

Eating disorders (ED) are serious mental health conditions that affect a person physically and psychologically [1]. The Diagnostic and Statistical Manual of Mental Disorders (DSM)-5-Text Revised (DSM-5-TR) identifies four primary categories of eating disorders: Anorexia nervosa (AN), Bulimia nervosa (BN), Other specified feeding or eating disorder (OSFED), and Binge eating disorder (BED) [2]. The cases of EDs (41.9 million) are underrepresented as per the Global Burden of Diseases, Injuries, and Risk Factors Study (GBD) (2019) [3]. Individuals with eating disorders have significantly higher mortality; rates are particularly high (5–7 times) for those diagnosed during hospitalization [3]. In 2019, the Disability Adjusted Life Years (DALYs) for EDs were reported to be 6.6 million [3].

The treatment of EDs is complicated by the coexistence of other psychiatric disorders. Approximately 87–94% of adults diagnosed with an ED also meet the criteria for another mental disorder at some point in their lives [4]. The most common comorbid diagnoses in adults include mood disorders (54–79%), substance use disorders (60–67%), and anxiety disorders (40–59%) [4]. Individuals with eating disorders are at a higher risk of hospital presentations due to self-harm behaviors [5]. Additionally, the rates of completed suicide among those with ED are significantly elevated compared to the general population. Specifically, individuals diagnosed with AN are reported to be 18 times more likely to die by suicide, while those with BN are seven times more likely to die by suicide [1]. In the past, EDs were only recognized as a western phenomenon, however, research evidence also confirms its presence in non-western countries including south Asia [6], though the evidence is very limited from Low and Middle Income Countries (LMICs), including Pakistan. There is a need to develop clinical practice guidelines to detect EDs, particularly in young Pakistani females [7]. The overall prevalence of EDs in Pakistan is unknown, though a descriptive cross-sectional study indicated 22.75% individuals to be at high-risk of EDs, with 87.9% females and 12.1% males [8]. Another study found 35.9% of students were at high risk of eating disorders according to the EAT-26 questionnaire, with 48.9% at high risk according to The Sick, Control, One, Fat, Food (SCOFF). The prevalence was higher in females and those with normal Body Mass Index (BMI), suggesting, early detection and better treatment are crucial for full recovery, also highlighting the need for targeted strategies in this population [9]. These rates cannot be generalized to the whole population as both studies were conducted in only one city. Moreover, negative weight perceptions, and concerns about body image in individuals with EDs are profound among Pakistani population [10].

A relatively recent systematic review provides a strong basis for the effectiveness of Cognitive Behavioral Therapy (CBT) as a psychosocial intervention in the treatment of EDs [11]. A CBT based guided self-help manual *“Getting Better Bite by Bite: A Survival Kit for Sufferers of Bulimia Nervosa and Binge Eating Disorders”* *(GBBB)* [12], has been found to be feasible and acceptable for patients with BN in Japan [13]. This intervention consists of 16 weekly sessions and has been tested widely in clinical trials [14]. To the best of our knowledge the evidence on psychological management of EDs in LMICs is limited, and it is non-existent in Pakistan. Therefore, the aim of this mixed method feasibility Randomized Controlled Trial (fRCT) is to explore the feasibility and acceptability of therapist delivered, manualized culturally adapted-GBBB (Ca-GBBB) intervention for individuals who screen positive on measures of BN and BED in Pakistan.

### Objectives

The objectives of the proposed study are:

- To train master level psychologists in delivering Ca-GBBB intervention.
- To determine whether the intervention is acceptable for individuals who screen positive on measures of BN and BED and therapists delivering the intervention.
- To evaluate the feasibility of planned intervention.
- To identify barriers and facilitators of successful delivery of the intervention.
- To determine most suitable outcome measures for future RCTs.
- To gather preliminary data for the primary outcome measure to conduct a sample size calculation.

## Methods

The proposed study will be a mixed-method fRCT of a therapist delivered, manualized Ca-GBBB intervention for individuals who screen positive on measures of BN and BED in Pakistan.

### Randomization and masking

The trial statistician will randomize participants (1:1) into either of two arms (1) the intervention arm; in which participants will receive Ca-GBBB along with routine care or (2) the routine care arm; in which participants will continue to have access to their routine care. The randomization will be performed using a computer generated algorithm, through block randomization to ensure an equal distribution of participants between groups. The researchers completing follow-up assessments will be masked to treatment allocation.

### Setting

The study will be conducted in five sites across large cities in Pakistan: Karachi, Hyderabad, Lahore, Rawalpindi, and Multan. Participants will be recruited from hospitals (i.e., the psychiatric department of the public and private hospitals, and medical wards), fitness centers (i.e., gyms), educational institutes (i.e., colleges and universities) and community settings (i.e., community health clinics). Self-referrals from general practitioners and community health workers will be accepted.

### Sample size

We are aiming to recruit a total of N = 80 participants, with 40 participants randomized to each treatment arm. The assessment of the sample sizes for the pilot and fRCTs indicated that the median sample size per arm across all types of studies was 30 [15]. Although we are not expecting a large drop-out rate in this study, we aim to randomize 80 participants.

### Participants

#### Inclusion criteria for participants

1. Age18 years and above.
2. Screen positive for BED on the Binge Eating Disorder Screening Questionnaire (BEDS-7) (cutoff score is the total score of ≥ 5) and/or BN on the Bulimic Investigatory test, Edinburgh (BITE) (cutoff score is the total score of ≥ 15).
3. Living in the catchment area of the study.
4. Capable of providing informed consent.

#### Exclusion criteria for participants

1. Below age 18.
2. Comorbidity of any other serious physical (any physical or medical condition reported by the potentially eligible participants will be documented at the time of screening and discussed with the study clinician to determine the participant’s ability to participate in the intervention and assessments) or mental illness (such as psychosis, bipolar disorders etc.) that could prevent individual from participating in the study (assessments or intervention).
3. Individuals who are unlikely to be available for sessions or follow-up due to temporary residency in the study recruitment areas will not be included in the study.

### Recruitment

- The study recruitment flyers (advert) will be circulated at participating health centers, and potential participants will have the opportunity to contact the researchers if they are interested in taking part or want to know more about the study.
- It will be made clear at this stage that agreeing to speak to a researcher does not mean they have to take part, and they can change their minds at any point.
- Researchers would use either an official landline phone or a research mobile phone explicitly bought for use within the project. No personal phones will be used to contact potential participants or participants. Likewise, only official email accounts will be used to contact participants via email.
- Individuals who express an interest in the study or wish to participate will then be approached by a researcher either by telephone or in-person (where telephone contact is not possible, and a face-to-face visit is deemed more practical). This will be important to ensure the person does not feel coerced into the research and has the opportunity to ask questions. The study (objectives, risks, and benefits) will be explained to the interested person, and the Participant Information Sheet (PIS) will be provided.

### Procedure

**Fig. 1:**
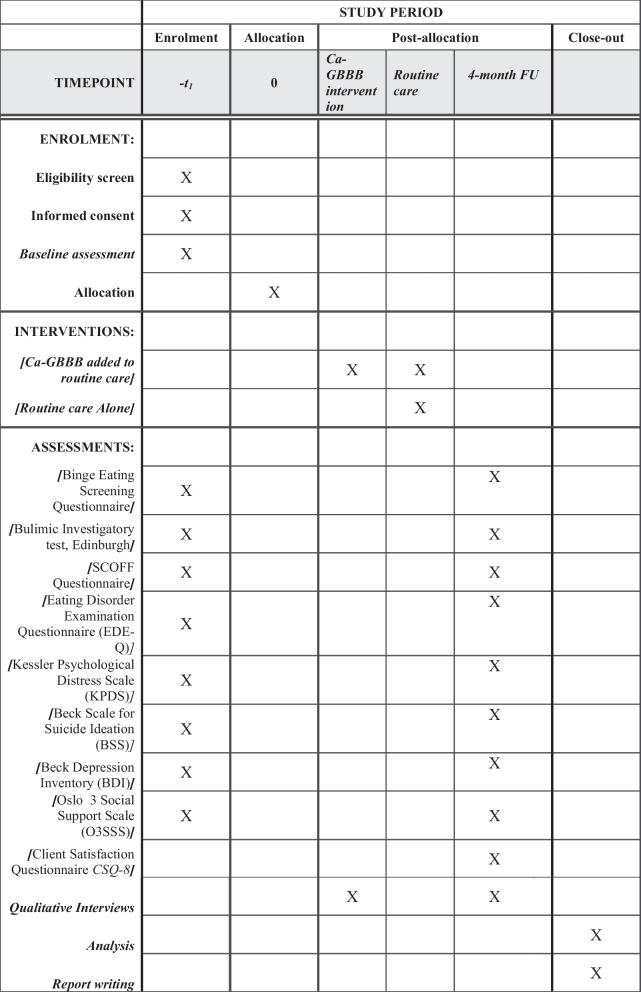
Trial flow chart

Ethics permission for the trial was sought from the National Bioethics Committee (NBC) of Pakistan (Ref: No.4-87/NBC-669/21/400). The trial is registered at clinicaltrials.gov (NCT05724394). Individuals who wish to participate in the study and are ready to sign informed consent will complete the consent process for screening (see Fig. 1 Trial Flow Chart). A trained researcher will assess the eligibility of the participants by using the study eligibility checklist. As part of eligibility assessment, the researchers will administer the BEDS-7 to screen for BED and the BITE to screen for BN. Eligible participants will be given a PIS and the purpose of the trial will be explained. All potential participants will have the opportunity to ask for more details about the study. Written consent will be obtained from all eligible participants who are willing to participate. Baseline assessments of consented participants will be conducted by the researchers trained in the Good Clinical Practice (GCP) who will administer study questionnaires. Assessments will be completed either face to face at a research office or recruiting health facility, or online over a telephone call or via Zoom link, depending on participant’s convenience. After completing baseline assessments, participants will be randomized into one of the two trial arms. Participants will be informed about the status of their allocated study arm within 1 week of randomization. Participants in the intervention arm will receive sessions of Ca-GBBB for a period of 16 weeks. Follow up assessment will be completed of all the participants regardless of their treatment arms at 16th week post-randomization. The assessments will take 45–60 min to complete.

### Intervention

#### Culturally adapted Getting Better Bite by Bite (Ca-GBBB)

The Ca-GBBB is based on the principles of Cognitive Behavioral therapy and evidence-based strategies for successfully changing problem behaviors as outlined in the National Institute of Clinical Excellence (NICE) guidelines for Behavior Change. The Ca-GBBB places a strong emphasis on improving participants' motivation and self-efficacy to facilitate change (Please see Table 1).

**Table 1:** Intervention session summary (Ca-GBBB)

Chapter 1: The Way Forward	Five years from now Do I suffer from bulimia? 1. Getting to know about bulimia and its severity is discussed 2. Bulimic investigatory test (self-assessment) Reasons to give up bulimia (Thought Restructuring) 1. Practical gains and losses for self 2. Practical gains and losses for others 3. Emotional gains and losses for self 4. Emotional gains and losses for others Advantages of change and recovery Activity: Letter for hope 1. Writing a letter to your friend Making your decision: Setting the goal 1. SMART goals
Chapter 2: Tools for the Journey	Facilitate change by keeping a therapeutic diary 1. Diary writing habit 2. Supporting role of diary Understanding Behavioral Chain: ABC 1. Getting familiarity of your eating disorder by analysing it with ABC approach A = Antecedents (triggers) B = Behavior C = Consequences New skills to cope with old difficulties 1. Seven steps approach
Chapter 3: Dieting: A Health Warning	Beauty is in the eye of the beholder 1. Standards of beauty varies with time 2. Body weight according to height What weight is right for me? 1. Weight and shape by physical constitution 2. Gene inheritance 3. Weight fluctuations Health Hazards of dieting 1. Dieting is dangerous 2. Effects of starvation on body and mind How much do I need to eat? 1. Achieving optimal weight and shape 2. Deciding to eat which depends on your metabolism rate and amount of energy consumed 3. Making a start (planning for a healthy diet plan) Regaining the eating control 1. What, when and how much to eat?
Chapter 4: The black hole of the insatiable stomach	Discussion on Bingeing 1. Case Examples Why can’t I control my eating? 1. Biological reasons 2. Psychological reasons How to stop binges 1. Planning meal time and pattern of eating 2. Dealing with psychological factors of bingeing 3. Managing cravings of food Coping with cravings and urges 1. Detachment 2. Imagery 3. Logic 4. Distraction Lapses: what to do if you relapse?
Chapter 5: Having your cake and eating it too	Facing the facts 1. Facts about effectiveness of weight control methods 2. It is right to worry Type of weight controller Tips to take a start 1. Planning how to start and adhere to the plan 2. How to cope with anxiety 3. Golden rules How to stop abusing laxatives, diuretics, medications 1. Coping with constipation 2. Coping with swelling
Chapter 6: Learning to feel good about your body	Learn to accept your body 1. How body image problems are caused and maintained 2. The role of media and social media 3. The perils of selective attention Getting to know your body Looking after your body Living with your body Other helpful strategies
Chapter 7: Being fatter may be better	Health risks in being overweight 1. Explained with examples Nothing is beautiful about dieting 1. Explained with examples Avoiding the lonely trap 1. Explained with examples 2. Importance of exercise Overcoming obstacles 1. How to deal with problems of eating disorder 2. Strategies to include in your lifestyle
Chapter 8: Relapse: Walking in circles—or not	Preventing slips 1. Plan your own relapse 2. Relapse Plan What to do if a slip occurs 1. Self-compassion 2. Self-kindness 3. Being aware of your mistakes Add pleasure to your day 1. Learning from slips 2. Chart activity
Chapter 9: Childhood Wounds	Sexual abuse 1. What is sexual abuse? 2. How do I know if I have been sexually abused? 3. Why is it wrong? Trying to make sense of it 1. Feelings of victims of sexual abuse 2. Explain with example 3. The right to be angry 4. What can you do with angry feelings? Grappling with guilt and self-blame 1. Identification of victims’ feelings of guilt and self-blame 2. Activity After-effects of abuse 1. Focusing on surviving Get a toehold on trust 1. Building trust on self and others Coming to terms 1. Accepting the past 2. Working out the need of counselling and therapy
Chapter 10: Food for Thought	Feeling like you don’t fit in 1. Eating disorder origination from childhood 2. Explain with example 3. Self-Defeating thoughts The gloom-and-doom scenario 1. Failures are due to your own doing or personality 2. This will always be the case in all situations When life is dreadful 1. Life is not good and everything will go wrong 2. Explain with examples 3. APT (Awareness, Planning and Try it) framework Wracked by guilt 1. A powerful sense of having done something wrong accompanies many sufferers of eating disorders in their daily life 2. Guilt is a hard problem to tackle Please, please them 1. The plastic pleaser 2. The bitch untamed 3. How to escape the pleasing pit When control gets out of control 1. People with eating disorders often organize their thinking by aiming for total control 2. People with eating disorders have both an innate and a learnt tendency to over-control 3. Explain with example 4. The ace of self-denial 5. The semblance of power Defusing self-defeating thoughts 1. Checking thoughts rational or irrational? 2. Identify thinking pattern 3. ABC of thoughts and diary writing 4. APT strategy for plan of action Shoo away shame 1. Feelings of shame, embarrassment and humiliation are closely linked to the belief that you must be perfect or else nobody will like you 2. To defuse these feelings, think of something you can do that is deliberately “not perfect” 3. Defy the tug of perfectionism and exult in the freedom that this brings
Chapter 11: Finding your voice	Learning to stand your ground 1. Learning about ways of communication (passive, assertive and aggressive) Anything for a quiet life 1. Disadvantages of not voicing your needs, wants and feelings Ground rules for assertive behavior 1. Basic human rights 2. Prior preparation and planning 3. Employ other techniques if being baited by criticism Putting assertiveness into practice 1. Practise what you want to say in front of the mirror 2. Record what you want to say 3. Role-play the situation with your recovery guide or another friend 4. Change roles, take on the person to whom you make the request The slippery slope of alcohol and drugs 1. Case examples 2. Designer drugs 3. Caffeine and artificial sweeteners When to worry about alcohol intake 1. The safe limits 2. Have the guts to stop or drink less 3. How to cut down Living dangerously 1. Development of bad habits like shoplifting and overspending to deal with bingeing 2. Explain with case examples Spending what you don’t have 1. Explain with case examples 2. Overspending or shoplifting—breaking the habit through ABC technique
Chapter 13: Web of life Parents, partners, children and friends	At home with the family 1. Improving your relationship with your parents 2. Explain with case examples Friends 1. With a little help from my friends 2. Making friends Sexual relationships 1. Frightened of sex 2. The wrong man 3. Promiscuity Children 1. Can I get pregnant? 2. Could I damage my baby? 3. What will happen to my eating disorder during and after pregnancy? 4. Will I cope with gaining weight during pregnancy? 5. I am worried about being a bad mother
Chapter 14: Working to live, living to work	Common work problems 1. Don’t have a job 2. Don’t have a right job 3. Not right for the job Gain and losses for myself Gain and losses for others Self-approval and disapproval Approval and disapproval from others
Chapter 15: The end of your journey – or not	If you are still stuck Time to get real about you Recovery: An adventure in self-discovery
Chapter 16: Where to get help and support	Worldwide eating disorder advocacy organizations Books and other resources Further reading

Participants’ convenience will be considered while deciding the time and venue of the sessions. Intervention will be delivered in weekly sessions over four months, and each session will last for 45–50 min. Special consideration will be given to the cultural adaptation of the content i.e. phrases of the manual while translating it into Urdu. Moreover, culturally relevant cases will be integrated, and a shared understanding will be aimed to address cultural aspects (gender roles and financial challenges etc.).

#### Training and supervision

The intervention will be delivered by master level psychologists who are being trained by the expert (ZZ) and monthly training refreshers and role plays will continue throughout the intervention period of the study. The training sessions involve a detailed presentation on content of each session, followed by group discussion, role play, feedback, and question answer session. The supervision plan includes fortnightly supervision meetings of each therapist with designated supervisors (ZZ, TK) that will include case presentations followed by discussion on any challenges experienced by the therapist.

#### Routine care

In Pakistan, standard routine care is provided by the local medical, psychiatric, and primary care services. Participants will receive a baseline evaluation along with routine care as determined by their treating physician. As part of the safety protocol, we will obtain the contact details of the participants’ GP. Details of any treatment received by all the participants will also be recorded in this study. Researchers delivering the interventions will have no contact and will not be involved with the participants in the routine care arm.

#### Cultural adaptation of GBBB

For cultural adaptation of the intervention manual, the content in the manual was translated by a team of bi-lingual researchers (fluent in understanding and speaking English and Urdu). The translated version was cross-checked by two researchers, independent of the first round of translation, to check the accuracy of translation. All the members of translation team were psychologists, making sure that they were familiar with the terms used in the English version of the manual. Translation was followed by the cultural adaptation by a multi-disciplinary team (psychologists, psychiatrists, nutritionists, representatives from the Community Engagement and Involvement group, CEI group). The multi-disciplinary team met weekly for 8 weeks and discussed the content of each session in detail to assess the cultural appropriateness. The adaptations were made on:

- Surface level (culturally relevant names and places)
- Deep level (culturally sensitive aspects alcohol consumption, going to pubs, living relationships etc.).

The manual adapted by this team was then discussed with CEI group. The CEI group supported to make further refinements in terms of using easy Urdu words and making worksheets more engaging for the intervention participants.

### Feasibility measures

#### Feasibility of the study procedures

We will operationalize feasibility through the question, ‘Can it work?’ [16]. The study will consider two main parameters: the recruitment rate, which will record the proportion of eligible patients with BED and/or BN disorder referred from and approached at the recruitment sites, and the attrition rate, which will track the number of patients who withdraw from the study after providing their consent to participate. To determine feasibility, the success criterion will be to recruit more than 50% of eligible participants. We will also explore, through researchers’ log, whether administering the assessment questionnaire is feasible (i.e. Can validated Urdu-language outcomes measures be obtained and administered?) and whether researchers can appropriately administer the assessment questionnaires. In addition, researchers will also document any feedback from participants. Feasibility of the randomization and blinding procedures will also be assessed.

#### Feasibility of the intervention and delivery

This will be evaluated by keeping record of whether the Ca-GBBB is delivered, received and enacted as intended. We will also keep records of each session duration, and collect feedback about administration and suitability of the intervention components for each session through a feedback form.

#### Acceptability and tolerability of the intervention

Acceptability is defined as “the extent to which the therapist delivering the intervention and the participants receiving the intervention consider it to be appropriate” [17].

Tolerability is defined as “the ability to endure the intervention” [18].

These aspects will be assessed as participant satisfaction with the Ca-GBBB intervention that is operationalized by the:

(i) Record of the number of sessions completed by each participant (*The criterion for acceptability is a mean attendance rate of* > *50% i.e. at least 8 sessions*).
(ii) Total duration of each session.
(iii) Participants’ feedback on the assessment and intervention maintained through the feedback forms and therapist session logs.
(iv) Participant satisfaction with the intervention assessed with the Client Satisfaction Questionnaire (CSQ) [19].
(v) In-depth semi-structured interviews with participants completing the intervention and participants not attending the session.

### Outcome assessment measures

**Fig. 2:**
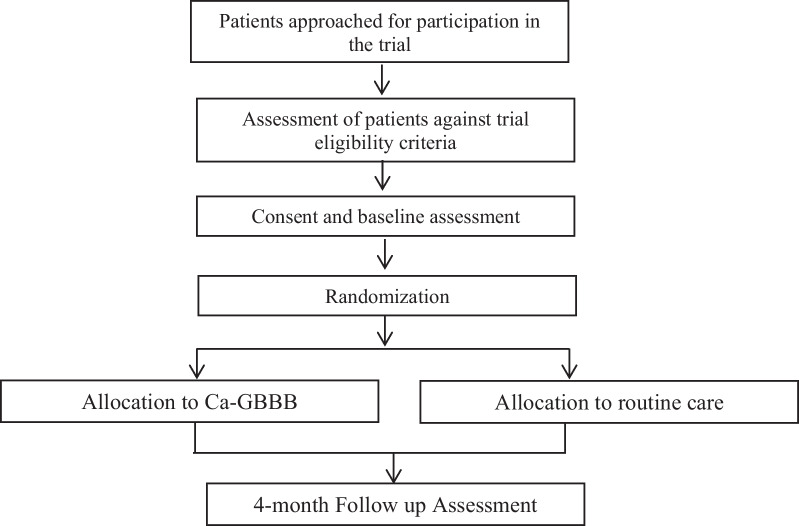
SPIRIT flow diagram

The following assessments will be completed at baseline and 4-month post-randomization (i.e., end of intervention) (See Fig. 2 SPIRIT Flow Diagram).

The Bulimic Investigatory Test, Edinburgh (BITE): The BITE is a self-rating scale comprising 33 items [20], divided into two subscales: the Symptom Scale with 30 items, measuring the extent of symptoms, and the Severity Scale with 3 items, indicating the severity based on the frequency of binge-eating and purging behaviors. The Symptom Scale items are answered in a yes/no format. Scoring ≥ 20 on the Symptom Scale indicates a highly disordered eating pattern and the presence of binge-eating, while a score of 10–19 suggests abnormal eating behavior like compulsive eating.

The Severity Scale, consisting of items 6, 7, and 27, investigates the frequency of fasting, purging, and binge-eating using a Likert scale. A score ≥ 5 suggests clinically relevant bulimic symptoms, and a score ≥ 10 indicates a high degree of severity. However, exceeding the Severity Scale threshold while staying within the normal range for the Symptom Scale does not lead to a diagnosis of BN; it may indicate the presence of psychogenic vomiting or laxative abuse without binge-eating.

The total score is obtained by summing the scores of both subscales. A total score > 25 indicates a potential case of binge eating. This questionnaire has been effectively used as a screening tool to identify binge-eaters in various countries [21].

Binge-Eating Disorder Screener (BEDS-7): The BED is a 7-item brief screening tool to screen Binge-Eating Disorder through 7 items [22]. The tool has previously been used on the Pakistani population, and the Cronbach's alpha coefficient obtained was 0.82 [23].

The Sick, Control, One, Fat, Food (SCOFF): The SCOFF [24] is a 5 item questionnaire consisting of three main domains of eating disorders which are (1) anorexia nervosa, (2) bulimia nervosa, or (3) other eating disorders. Each item marked as “yes” gets a score of 1 and summed up to get a cumulative score. Originally, a cut-off point of 2 or greater was established to achieve maximum sensitivity in detecting anorexia and Bulimia nervosa [24]. However, it has been recommended that a cut-off point of 3 offers the best balance between sensitivity and specificity. The tool has previously been used on the Pakistani population, though no Cronbach's alpha coefficients for the Pakistani population were determined [9, 25–27].

Eating Disorder Examination Questionnaire (EDE-Q): It’s a 28-item self-reported questionnaire adapted from the semi-structured interview to examine Eating Disorder. It is designed to evaluate the range and severity of features associated with a diagnosis of eating disorder using 4 subscales (Restraint, Eating Concern, Shape Concern and Weight Concern) and a global score. A cut-off of 4 on the global score is generally considered as clinically significant [28]. The scale has already been used on the Pakistani population, but the reliability coefficient was not calculated [25].

Kessler Psychological Distress Scale (KPDS): This 10-item questionnaire aims to assess psychological distress. The scale measures the emotional states with a 5 level response scale [29]. It has proven to be a reliable tool to administer to the Pakistani population as literature shows high reliability (0.89) for K-10 among the Pakistani Population [30].

Beck Scale for Suicide Ideation (BSS): To assess the frequency and intensity of suicidal thoughts, 21 item scales for suicidal ideation was developed by the Beck [31]. Each of the 19 items in the BSS is scored on a scale from 0 to 2, resulting in a total score ranging from 0 to 38, where higher scores indicate increased suicidal ideation. The BSS demonstrates favourable psychometric properties [31]. It has been used in the Pakistan previously [32], and the reported Cronbach's alpha for the Urdu translated version of the BSI were 0.75 [33] and 0.89 [32].

Beck Depression Inventory (BDI): The 29 item questionnaire of BDI [34] will be used to measure severity of depression. Score range of each item is 0–3 whereas severity ranges from minimally depressed (score lower than 13) to severely depressed (scores between 29 and 63). It is a globally validated tool to assess depression. The Urdu translation of the instrument showed a high Cronbach's alpha of 0.96 [32].

Oslo 3 Social Support Scale (O3SSS): O3SSS [35] will be used to assess the relationship with friends, family and neighbours. Each item in the instrument has a 5-point rating scale, resulting in a total score ranging from 3 to 15, where higher scores show stronger levels of support. The instrument has undergone validation in Urdu [36], and Cronbach's alpha for the Urdu version was 0.46 [37].

Client Satisfaction Questionnaire (CSQ-8): The CSQ will be used to assess the participants’ satisfaction with services received during the study [19].

### Qualitative component

A purposefully selected subset (stratified by age & gender) of participants (N = 15) in the intervention group will be invited to participate in a qualitative interview to explore their experiences and satisfaction with the intervention. Through a purposeful selection, we want to make sure that participants from all age brackets (both adults and middle-aged) and from both genders can participate in the qualitative interview.

A senior qualitative researcher will select the participants who will be independent of participants’ recruitment, intervention delivery, and response to treatment. The qualitative researcher will not have any information about participants' scores on assessment questionnaires and their responses to the intervention. This means that they will only be aware of basic demographic characteristics and not the assessment results. On average, interviews will last for 60 min.

The interviews will be either face-to-face or through telephone at a mutually agreed upon place and time by both the researcher and the participant.

- The interviews will be guided by a semi-structured topic guide. The topic guide will explore participants’ experience of participating in the intervention sessions including their feedback on content of the intervention and activities completed during sessions, perceived usefulness of the intervention, suggestions to improve the experience of participating in the intervention, feedback for therapists who delivered the intervention, challenges, and facilitators of participation in the intervention (Please see Appendix 1).
- All interviews will be audio recorded with the prior consent of the participants.

### Quantitative analysis

This is a pilot/feasibility study, therefore, the proposed study is not powered to detect between group differences. Our main outcome is the recruitment and retention of potential participants into the study. Consequently, we will report the recruitment and retention rates. Moreover, since the feasibility study does not aim to test differences or compare groups with formal hypothesis testing, we will report the outcome measures at both baseline and follow-up using appropriate descriptive statistics, measures of variability, and confidence intervals. Once the primary outcome measure for the full trial has been identified we will use the information provided in this study and a clinically meaningful difference to determine the effect size and corresponding sample size for the full trial. For this purpose, multiple measures of similar constructs are proposed to be administered where possible (e.g., eating disorders) to determine which measure to include in the definitive trial according to feasibility, acceptability, and sensitivity. In addition, the correlation of scores on different measures with severity of BED and/or BN will provide information about which measures are sensitive to change or congruent with the conceptual causal model of the intervention.

### Qualitative analysis

We will use the framework analysis approach proposed by [38] for analysis of qualitative data [38].

*Stage 1*: Transcription: All the audio recorded interviews will be transcribed verbatim by the master level psychologists with previous experience of transcription. Non-disclosure agreements would already be in place for these researchers. The qualitative researchers will randomly cross check the transcripts against the audio recording to ensure the accuracy and quality of the transcription.

*Stage 2*: Familiarization with the data: The qualitative researchers will read and re-read the transcripts and the reflective notes recorded by the interviewers to fully immerse themselves in the data set. They will also re-listen to the audio recording if needed. They will make their analytical notes, thoughts or impressions during the stage of familiarization.

*Stage 3*: Coding: The researcher, while reading the transcripts line by line, applies a label (a ‘code’) that would describe what they have interpreted in the passage as important. Coding will aim to classify all of the data so that it can be compared systematically with other transcripts. Two researchers will independently code at least 25% of the transcripts. Coding will be done manually with a paper and pen.

*Stage 4*: Developing a working analytical framework: After coding the 25% of transcripts, both researchers will meet to compare the labels they have applied and agree on a set of codes to apply to all subsequent transcripts. Codes will be grouped together into categories, which will be then clearly defined. The analytical framework will not be ‘final’ until the last transcript is coded.

*Stage 5*: Applying the analytical framework.

The working analytical framework will then be applied by indexing subsequent transcripts using the existing codes and categories.

*Stage 6*: Charting data into the framework matrix.

To manage and summarize the data, an excel spreadsheet/Nvivo will be used to generate a matrix, and the data will be ‘charted’ into the matrix. Charting involves summarizing the data by category from each transcript. Good charting will allow us to strike a balance between reducing the data on the one hand, and retaining the original meanings, and ‘feel’ of the participants’ words, on the other. The chart will include references to illustrative verbatim.

*Stage 7*: Interpreting the data.

Characteristics of and differences between the data will be identified, perhaps generating typologies, interrogating theoretical concepts (either prior concepts or ones emerging from the data) or mapping connections between categories to explore relationships and/or causality.

### Community Engagement and Involvement (CEI)

A Community Engagement and Involvement (CEI) group has been established to advise the research team during the development of research protocol. This group is comprised of five members and one CEI lead with lived experience. A detailed CEI plan was developed by the CEI lead in consultation with the CEI members. This CEI plan was discussed in a wider Community Engagement and Involvement Group and suggestions were incorporated. The CEI group has advised on the areas that should be assessed during the study, development of study material such as participant information leaflet, interview schedule, study adverts, and data collection questionnaires. The CEI group actively participated during the adaptation of the intervention. The research team will continue to engage with this group to develop robust recruitment and retention strategies, analysis of qualitative findings and development of dissemination material. The CEI group will also contribute to the community engagement activities.

### Communication plan

A comprehensive communication plan will be developed in collaboration with CEI group and other stakeholders by taking into consideration the culturally appropriate strategies to share the purpose and need of the project as well as the study findings. Partnership with the community has already been established as part of completed and ongoing suicide prevention work in Pakistan (CMAP MR/N006062/1; YCMAP MR/R022461/1; SEPAK GCRF Pump priming award; and SAHAR-M GCRFF006949). We will extend and further strengthen this network through engagement meetings with stakeholders, potential voluntary organizations, local health teams, primary care settings, GP practices, hospitals, and community centers in order to increase awareness and promote the study. We will use local mainstream and social media channels, press releases, posters, newsletters and information leaflets in English, Urdu, and other local languages, and clinical and local community settings to advertise the study to a wider audience. In addition, we will also organize community awareness workshops, seminars (through zoom or face to face) in catchment areas of the study with a particular focus on the low income and low literacy population. The results of the study will be written up for academic publications in peer reviewed, open access journals and in national and local newspaper articles in lay language. The results will also be further disseminated through presentations in international conferences, local and national TV and radio channels.

### Safety

As this study involves vulnerable people experiencing mental health difficulties, a safety protocol will be in place. The research team will receive regular ongoing supervision for managing distressed populations by an expert psychiatrist (NC), and an experienced clinical psychologist (TK). In case of any adverse event (i.e., suicide attempt or self-harm), an Adverse Event Form (AEF) will be completed and sent to the PI within 24 h of reporting of the event. The Project Management Committee is led by the senior consultant psychiatrist. This committee will meet on weekly basis to monitor the study progress and safety of the participants. Data management plan will be developed and adhered to. The identifying information will be kept in locked cabinet with access to authorized research team members. The audio-recordings of interviews will be saved in password protected computers.

## Discussion

The proposed study will provide evidence on feasibility and acceptability of a culturally adapted manually assisted psychological intervention for individuals who screen positive on measures of eating disorders. The findings of the study will provide an opportunity to plan and design a fully powered randomized controlled trial to evaluate clinical and cost-effectiveness of Ca-GBBB. Both the quantitative, and qualitative results of this feasibility study may contribute to improve clinicians and researchers’ understanding on what is likely to be effective for individuals with eating disorders. In LMICs, particularly Pakistan, there remains a substantial deficit in evidence on management of EDs, and this study will be the first trial evaluating the feasibility of the culturally appropriate psychological intervention for this population.

The study will produce the following outputs.

- A user-friendly, culturally adapted, Urdu translated manual of Ca-GBBB.
- A training tool kit for students, researchers, academics and clinicians.
- Simple awareness leaflets in national and regional languages.
- Publications in peer reviewed, open access journals.
- Lay summaries of findings ready to be disseminated through different platforms including social media.
- A dissemination plan for the study will be prepared in collaboration with the CEI group. The findings will be presented in local, national and international conferences.

## Data Availability

The anonymized data will be available upon request by email to the corresponding author.

## Appendix 1: Topic Guide-Participants

**Instruction to interviewer:** The following interview schedule serves as a guide. Whilst the interviewer should endeavour to cover the content of the interview schedule, precise wordings and questions may be varied as required.

**Instructions to participant:**

We are meeting today to ask you about your experience of participating in the intervention programme, including your thoughts about any barriers or challenges that you may encountered during participation. This interview will help us to improve this intervention programme.

**Introduction, setting ground rules:**

Introduce yourself, thank participant for taking part and confirm agrees to interview taking place.

Ensure environment is comfortable.

Discuss the following issues:

- Review the nature and purpose of the research.
- No right or wrong answers, aim to understand experiences/views.
- Confidentiality, use of data.
- Explain the use of data recorder, transcription, use of pseudonym (invite to choose), use of verbatim quotes, will be taking field notes.
- Researcher should understand that  discussion might bring up difficult memories, explain that the participant can decline to answer any question or prompt; they can ask to stop at any time if feels need to.
- Expected duration of interview.
- Check consent form signed.
- Ask if any questions.

Check recorder working.

Introduce and switch on tape recorder.

General prompts.

- Allow participant to respond uninterrupted and use open prompts if required to explore aspects of their experiences in depth.
- Can you tell me more about XXX?', 'What makes you say XXX?, How did XX make you feel?

## Start of interview

I’m going to ask you first about your experience of the training/intervention program.

1: Could you please share your experience of participating in sessions? How was it like for you?

**Prompts:**

- What did you expect from this sessions/intervention? Was the program able to meet your expectations? if yes, can you please explain? if No, can you please talk more about this?
- What was helpful? and why?
- What was not very helpful? and why?
- Easy or difficult to understand?
- How was it getting to the sessions? (e.g. remembering, transport, time)

2: Can you share your experience about the topics you discussed during your meetings with therapists?

**Prompts**

- Psychoeducation about Eating Disorders
- Making your decision: Setting the goal
- Psychoeducation regarding body shape
- Health Hazards of dieting
- Letter for Hope
- Learning to Detect Early Symptoms of Relapse
- Understanding behavioural chain: ABC Model
- How to stop binges
- Childhood Wounds
- Putting assertiveness into practice
- Working to live, living to work

3: Did the trainer/therapist ask you to do anything in sessions (activities)? What was this like?

**Prompt:**

- What did they ask you to? (5P’s, Reasons to give up bulimia, diary writing, seven steps approach, Coping with cravings and urges etc.)
- Why do you think you were asked to do this?
- Was it helpful or not?
- Have you experienced any problem doing these activities during the sessions?
- Did the trainer asked you to do anything in-between the sessions (homework assignment)? What was that? Was it helpful? Any challenges in completing homework tasks?

4: Have things in anyways changed for you after participating in these sessions? If yes, how? What aspects/components of these sessions helped to achieve these changes?

**Prompts:**

- Any changes in level of stress?
- Knowledge about your illness
- Attitude towards dieting
- Eating Control
- Use of laxatives, diuretics, medications
- Acceptance of body shape
- Communication style
- Relationship with family members
- Functioning

Are these changes for the better or for the worse?

Please tell me more about what is different now.

5: Any part of the sessions/intervention you are not happy with? Any aspect, that you think, should not be the part of these sessions? If yes, why?

**Prompts:**

- Was that stressful?
- Very personal?
- Difficult to understand?
- Not relevant for you?

Lengthy/time consuming?

6: Are you satisfied with the therapist/interventionist? Delivery format? Frequency?

**Prompts:**

- Attitude
- Selection of words? Was that easy or difficult to understand
- Tone of interventionist?
- Was he/she able to calm you down when at any point during the session you got stressed out?
- If you ever get worried or upset, will you prefer to come to the therapist/interventionist again?
- Did the therapist/interventionist give you enough time?

7a: Have you faced any challenges while participating in these sessions?

**Prompts:**

Time constraints.

Place of session.

Transport issues.

Family pressure.

Individual barriers (motivation etc.)

7b: What helped you to participate and attend these sessions (Facilitators)?

**Prompts:**

- Personal motivation
- Family support
- Availability of resources
- Benefit from the intervention
- Quality of relationship with interventionist/therapist

8: If you could change the training, what would you change?

Prompt: How would you like the training to be different? (in terms of format, content).

**Questions relating to intervention sessions missed by the participants (only relevant if participant missed sessions).**

I understand that you missed [number of missed sessions] sessions. This is not unusual and can happen for all sorts of reasons. I would like to ask you a few questions about this if that is okay?

9: What were the reasons for missing the session?

**Prompt**:

1. Was there anything about the training that meant you missed the session/finished early?
2. Was it anything to do with the trainer?
3. Were there other concerns or worries or relapsed that meant you missed the session/finished early?
4. Is there anything that could have been done differently that would have helped you stay in the training?

**Thank the participant. Ask about their feelings after participating in the interview, anything else that participant may want to talk about.**

## Abbreviations

ED: Eating disorders
DSM-5-TR: Diagnostic and Statistical Manual of Mental Disorders (DSM)-5-Text Revised
AN: Anorexia nervosa
BN: Bulimia nervosa
OSFED: Other Specified Feeding or Eating Disorder
BED: Binge eating disorder
GBD: Global Burden of Diseases
DALYs: Disability Adjusted Life Years
LMICs: Low and Middle Income Countries
SCOFF: Sick, Control, One, Fat, Food
BMI: Body Mass Index
CBT: Cognitive Behavioral Therapy
GBBB: Getting Better Bite by Bite
fRCT: Feasibility Randomized Controlled Trial
BEDS-7: Binge Eating Disorder Screening Questionnaire
BITE: Bulimic Investigatory test, Edinburgh
EDE-Q: Eating Disorder Examination Questionnaire
KPDS: Kessler Psychological Distress Scale
BSS: Beck Scale for Suicide Ideation
BDI: Beck Depression Inventory
O3SSS: Oslo 3 Social Support Scale
CSQ-8: Client Satisfaction Questionnaire-8
CEI: Community Engagement and Involvement
